# Nutritional features of organic peas (*Pisum sativum L*.) cultivated in different Italian environments and rheological profile of pea‐enriched crackers

**DOI:** 10.1002/jsfa.14156

**Published:** 2025-02-05

**Authors:** Camilla Tibaldi, Sónia Oliveira, Giovanni Dinelli, Ilaria Marotti, Anabela Raymundo

**Affiliations:** ^1^ Department of Agricultural and Food Sciences Alma Mater Studiorum – University of Bologna Bologna Italy; ^2^ LEAF—Linking Landscape, Environment, Agriculture and Food Research Center, Associated Laboratory TERRA Instituto Superior de Agronomia, Universidade de Lisboa Lisbon Portugal

**Keywords:** legumes, peas, PBMAs, nutritional composition, legume‐based crackers, rheology.

## Abstract

**BACKGROUND:**

Legumes are a key component of the human diet and a primary source of plant‐based protein. They have attracted global attention as potential plant‐based meat alternatives due to their numerous health benefits, and they contribute to a more sustainable and healthy food system. Among pulses, peas (*Pisum sativum* L.) are considered a good source of proteins, fibers, starch, minerals, and vitamins. This study evaluated the effect of environmental conditions on nutritional profile of peas cultivated in an organic farming system, in different Italian environments (mountainous and hilly), during different cultivation years (2021 and 2022). Pea grain from peas cultivated under the various conditions was used to prepare pea‐based crackers containing 6% pea flour. The appearance, physical properties (rheology and texture), and nutritional profile of the snacks were evaluated, and sensory analysis was conducted.

**RESULTS:**

The nutritional and bioactive compounds were strongly related and the environment exerted a substantial impact on most of the nutritional components (proteins and carbohydrates), due to climatic conditions during the vegetative and reproductive stage of the crop. The incorporation of cultivated peas into wheat‐based crackers improved their functional and nutritional quality while maintaining consumer acceptability, as demonstrated by sensory analysis.

**CONCLUSIONS:**

The results confirmed that growing conditions significantly influence the nutritional composition of peas, enhancing their quality and that of the resulting crackers. This aligns with the increasing global demand for high‐quality, sustainable food products. © 2025 The Author(s). *Journal of the Science of Food and Agriculture* published by John Wiley & Sons Ltd on behalf of Society of Chemical Industry.

## INTRODUCTION

The health and nutritional benefits of pulses and their by‐products have recently attracted significant attention from researchers and consumers, driving their cultivation and production to meet global demand and ensure high‐quality food. Pulses have a positive influence on human health, helping to reduce the risk of cancer, coronary heart disease, and diabetes.^1^, ^2^ Peas (*Pisum sativum* L.) are an important nutritional source of proteins, carbohydrates, resistant starch, dietary fibers, minerals, and vitamins.^3^ In addition to their nutritional value, they have increasingly attracted attention as a functional or nutraceutical food, due to the presence of secondary metabolite phytochemicals that present antioxidant, antimutagenic, and anticarcinogenic biological effects.^3^, ^4^

Increasing global awareness of environmental, human health, and food safety concerns has led to a growing focus on alternative protein sources to meat.^5^, ^6^ Indeed, recently, a new generation of plant‐based meat alternatives (PBMAs) has generated considerable consumer interest, as they are considered to be a sustainable substitute to meat‐based diets.^7^ Environmental studies have reported that PBMA production generates less greenhouse gas (GHG) emissions and requires lower energy, water, and land use than livestock production.^8^ Legumes are a good source of plant‐based proteins and may represent one of the largest sources of PBMAs. The production of legumes and pulses has recently increased in Europe.^9^, ^10^

With the new impetus focused on a more sustainable food system, the integration of legumes into the human diet may have significant health benefits, enhancing the nutritional and health properties of food products, and thus it may become a topic of interest to consumers and the food industry.^5^, ^11^ Baked snacks, such as crackers and biscuits, are usually well accepted and consumed throughout the world, and can be excellent vehicles for nutraceutical and protein enrichment because of their wide consumption and long shelf life.^12^ Previous studies have incorporated pea flour into wheat crackers improving their nutritional value.^13^

Legume proteins are rich in lysine but deficient in sulfur‐containing amino acids, whereas cereal proteins are deficient in lysine but have adequate levels of sulfur‐containing amino acids. Hence, the combination of cereal and legume proteins can provide a better overall balance of essential amino acids^14^ and may lead to beneficial diet effects by controlling and preventing various metabolic diseases, such as diabetes, cardiovascular disease, and some forms of cancer.^14^, ^15^, ^16^, ^17^

Given the importance of the nutritional quality of legumes, focusing on the impact of crop‐growing factors on the nutritional and functional properties of peas and pea‐based products could provide insights into environmental and agronomic practices that enhance the nutritional and physiological qualities of these legumes. The present study aimed to evaluate the nutritional composition of peas (*Pisum sativum* L.) cultivated under organic farming and low‐input conditions over 2 years consecutively in different environments within the Emilia‐Romagna region of Italy. The incorporation of organic pea flour into wheat‐based savory snacks (crackers) was also assessed in terms of appearance, physical properties (rheology and texture), and nutritional composition, with the goal of creating a high‐quality plant‐based product.

## MATERIAL AND METHODS

### Field trials

Open‐field experimental trials were conducted over 2 years consecutively (2021 and 2022) at two experimental locations within Emilia‐Romagna region, in mountainous (800 m altitude) and hilly (200 m altitude) environments. The sowing time differed depending on the growing environment and on the elevation of the sites: for the mountainous environment an autumn sowing was chosen (from November to July); for the hilly environment spring sowing (from March/April to July) was chosen for both years. At all locations, an organic farming system was followed: no pesticide or herbicide treatments were performed on the plants and the sites were all rainfed. Meteorological data (temperature and precipitation), for the entire duration of the experimental trial, comprising the multiple cycles in each location, was obtained from the Arpae weather station, located in Emilia Romagna (https://simc.arpae.it/dext3r/) and are shown in Table 1. Using the temperature data, the mean growing degree days (GDD) were calculated.^18^ With regard to the hilly experimental field, the trend in average temperatures appears to have been very similar between the two cropping years, although temperatures were higher in 2022. The two growing years differed in terms of rainfall patterns: in 2021, higher precipitation occurred in the month following sowing (April), whereas in 2022, rainfalls were significantly higher in the current month of the sowing. The same temperature and rainfall trends were observed for the mountainous experimental field. In 2022 total rainfall was more abundant during the vegetative and reproductive development of the crop (from February to May 2022) in comparison with the 2021 cropping year.

**Table 1:** Monthly precipitation, average temperature, and growing degree days, at each growing environment for all cultivation cycles, between 2021 and 2022.

	Rainfalls (mm)		Mean temperature (°C)		Growing degree days (GDD)	
	Hilly	Mountainous	Hilly	Mountainous	Hilly	Mountainous
2020						
Oct		91.50		13.4		9.4
Nov		30.1		9.1		5.1
Dec		175.4		5.0		1.0
2021						
Jan	43.8	68.4	2.1	3.6	−1.9	−0.4
Feb	14.6	17.8	6.6	7.3	2.6	3.3
Mar	30.8	15.6	8.8	9.2	4.8	5.2
Apr	61	59.9	10.6	10.8	6.6	6.8
May	42.2	30.5	16.3	15.7	12.3	11.7
Jun	23.4	18.2	23.8	23.5	19.8	19.5
Jul	8.6	17.7	25.5	25.1	21.5	21.1
Aug	15.6	27.9	24.7	24.3	20.7	20.3
Sept	73.8	91.9	20.6	20.7	16.6	16.7
Oct	28.2	39.8	12.9	12.8	8.9	8.8
Nov	111.2	123.9	8.3	8.6	4.3	4.6
Dec	54	59.3	3.4	5.0	−0.6	1.0
2022						
Jan	36.6	60	3.4	5.1	−0.6	1.1
Feb	14	39.3	6.2	7.8	2.2	3.8
Mar	41	57.9	7.5	7.7	3.4	3.7
Apr	91.8	92	12.3	11.3	8.3	7.3
May	27.2	78	20.1	18.9	15.9	14.9
Jun	11.8	16	24.9	24.0	20.8	20.0
Jul	23	9.2	26.9	26.4	22.9	22.4

*Note*: Meteorological data supplied by the Arpae weather station, located in Emilia Romagna (https://simc.arpae.it/dext3r/).

### Plant material

For each location, green ripening pea grain was harvested from 1 m^2^ of surface area within each randomized block. The pea biomass was stored at 4 °C and dried in air oven at 50 °C overnight, as described by Liu,^19^ the respective dry grain was milled (Billy 200, Hawos Mulini, Bad Homburg, Germany) to produce fine pea flour and stored at 4 °C until analysis.

### Pea nutritional characterization

#### Polyphenol and flavonoid content and antioxidant activity analyses

Polyphenols, comprising both free and bound constituents, were extracted as described previously.^20^ Free (FP) and bound polyphenols (BP) were then measured according to the Folin– Ciocâlteu spectrophotometric (765 nm) method using gallic acid (GA) as a reference standard.^21^ Likewise, the free (FF) and bound flavonoids (BF) were measured individually using a spectrophotometric (510 nm) colorimetric assay with catechin (CA) as a reference standard.^22^ The respective totals (TP and FT) were calculated from the free and bound polyphenol and flavonoids. The 2,2‐diphenyl‐1‐picrylhydrazyl (DPPH) assay was performed by measuring the reduction (515 nm) of DPPH• to 1,1‐diphenyl‐2‐picryl hydrazine^23^ and ferric reducing antioxidant power (FRAP) (reduction of Fe^2+^) was determined using a spectrophotometric (593 nm) method reported previously.^24^ The antioxidant activity in the free and bound fractions were summed and expressed as total DPPH and FRAP, respectively. All analyses were performed in triplicate.

#### Dietary fiber content

Insoluble dietary fiber (IDF) and soluble dietary fiber (SDF) were extracted and measured according to the instructions provided by the Megazyme Total Dietary Fibre Assay Procedure kit cat. no. K‐TDFR (Megazyme International, Bray, Ireland). This protocol was followed based on previously reported methods.^25^, ^26^ All analyses were performed in triplicate.

#### Lipid content

The Folch method was used for the determination of lipid content (AOAC 960.39, 1990).^27^ Briefly, 500 mg of pea flour was diluted with 1:20 in chloroform:methanol (2:1; *w/v*) and was shaken for 20 min. The tubes were centrifuged at 10 000 × *g* (10 min). The upper aqueous phase was transferred to a Büchner funnel and vacuum filtered into pre‐weighed beakers. After overnight hood drying, the beakers were weighed and the results were calculated as percentages of lipids. All analyses were performed in triplicate.

#### Protein content

The nutritional composition of the organic pea flour and crackers was investigated following the AOAC official procedures for baked products (AOC 950.36, 2006). The protein content was determined using Dumas protein/nitrogen analyzer VELP Scientific NDA 702 DUMAS Nitrogen Analyzer—TCD detector (VELP Scientifica Srl, Usmate Velate, Italy), according to the Dumas method. The total nitrogen content was determined, and the resulting value was multiplied by the legume protein conversion factor of 5.7 to obtain the crude protein content of the sample.^28^, ^29^ All analyses were performed in triplicate.

#### Digestible and resistant starch

Total digestible starch (TDS) and resistant starch (RS) were extracted and measured using the Megazyme Digestible and Resistant Starch kit cat. no. K‐DSTRS (Megazyme International). All analyses were performed in triplicate.

#### Sucrose, fructose, d‐glucose and raffinose family oligosaccharide content

Carbohydrates, including sucrose, fructose, and d‐glucose, were quantified according to the protocol provided with the Megazyme Sucrose/Fructose/d‐Glucose kit (cat. no. K‐SUFRG, Megazyme International). Similarly, raffinose family oligosaccharides (RFOs), specifically raffinose (RAF) and its precursor galactose (GLCT), were analyzed using the Megazyme Raffinose/d‐Galactose kit (cat. no. K‐RAFGA, Megazyme International). All analyses were conducted in triplicate.

#### Mineral composition

Organic pea flour (used to produce pea‐enriched crackers) was analyzed for its mineral elements by atomic absorption spectrophotometry and inductively coupled plasma optical emission spectroscopy (ICP‐OES) (iCAP 7000 series, Thermo Scientific, Waltham, MA, USA).^30^ All analyses were repeated three times.

### Pea‐based baked snacks

#### Cracker preparation

Savory snacks were prepared according to a previously developed and optimized model formulation using 65.5% wheat flour, 1% salt, 1.5% baking powder, 7.5% sunflower oil, and 24.5% water.^28^ A control cracker, with only wheat flour, was designed and analyzed. In the pea‐enriched samples, wheat flour was substituted with 6% of organic pea (*Pisum sativum* L.) flour, based on different studies on legumes‐incorporations in baked products,^29^, ^30^, ^31^ derived from the field experimentation. Batch sizes of 100 g were made, corresponding to approximately 30 crackers. All the ingredients were mixed by hand, using an optimized procedure, and then rolled out with a manual dough machine, reproducing the extrusion process (Atlas 150 Marcato, Campodarsego, Italy), to a thickness of 1.8 mm. The crackers were then molded into jagged 38 mm squares and baked at 180 °C for 5 min in a conventional oven (Johnson A60; Johnson & Johnson, New Brunswick, NJ, USA). After cooling, some crackers (*N* = 10) were powdered for nutritional composition and other chemical analyses.

#### Dough rheology

The viscoelastic behavior of the dough was investigated following a procedure described by Mota,^32^ using a controlled stress rheometer (Haake MarsIII, Thermo Scientific) equipped with a UTC–Peltier system to control the temperature. Frequency sweep tests were performed within the viscoelastic linear region, which was previously defined through a stress sweep test, at 1 Hz, using a serrated parallel‐plate geometry with a 20 mm diameter (PP20). Dough pieces were compacted to a 1.5 mm gap and the edge parts were coated with liquid paraffin to prevent moisture losses during tests. Stress and frequency sweeps were performed at 20 °C. The values of G' (elastic modulus) and G" (viscous modulus) were recorded as a function of oscillation frequency. The results were presented in terms of the mechanical spectrum, which provides insights into the internal structuring of the dough.

#### Color analysis

The color of the dough and the final products was measured using a Minolta CR‐400 (Japan) colorimeter. The method used was previously described by Mota.^32^ Measurements were conducted under consistent lighting conditions using a white standard (L* = 94.61, a* = −0.53, b* = 3.62) at a controlled temperature. Each sample (control and pea‐enriched snacks) was analyzed at least ten times, 24 hours after baking. Total color differences (*Δ*E) between controls and the pea‐enriched snacks were assessed using the following equation:

(1) $$\Delta E=\left(\Delta L^{*}\cdot 2+\Delta a^{*}\cdot 2+\Delta b^{*}\cdot 2\right)\cdot 1/2$$

#### Texture analysis

Texture analysis was performed with a TA.Xtplus texturometer (StableMicro Systems, Godalming, UK). The measurements were made at 20 °C. Each snack's texture was evaluated with a penetration test, using a cylindrical probe of 2 mm in diameter, plunged 10 mm at 1 mm s^−1^, as described by Mota.^32^ Hardness was measured as the peak force (N) on the force‐time texturogram, representing the maximum force required to break the cracker.

Crispiness was evaluated based on the time required to reach the maximum peak(s) in the texturogram. A shorter time to break indicates greater crispiness, as crispiness is inversely related to the time needed for breakage; the faster the break occurs, the crispier the cracker.^33^

These tests were reproduced at least eight times for each cracker (wheat‐based controls and pea‐enriched snacks) 24 h after baking.

#### Water activity determination

The water activity (a_w_) was analyzed using a thermos‐hygrometer (HygroPalm HP23‐AW, Rotronic AG, Bassersdorf, Switzerland) at 20 °C. After 24 h of baking, the tests were performed by crushing the crackers and each snack (wheat‐based controls and pea‐enriched snacks). The measurements were made in triplicate.

#### Cracker nutritional composition

The nutritional composition of the crackers was evaluated using the powdered samples. In accordance with the AOAC 950.36^34^ official method for baked products, the protein content was evaluated using the Dumas method, as described above. Crude fat was measured using ether extraction in accordance with AOAC 2003.05. Briefly, a minimum of 1.5 g of each snack (with and without pea flour) was weighed into a 26 mm × 60 mm cellulose extraction thimble. The petroleum ether lipid content was determined using a Soxtec extraction unit (Soxtec System HT 1043/1046, Tecator AB, Höganäs, Sweden). The procedure included 15 minutes of boiling, 60 minutes of rinsing, and 15 minutes of drying. Finally, the lipid content was determined gravimetrically. Ash content, representing the inorganic fraction of the snacks, was measured by incineration at 550 °C in a muffle (American Association of Cereal Chemists method 08–01.01). Moisture content was determined as described by Mota.^32^ Total carbohydrates were calculated by difference.

#### Sensory analysis

Crackers were evaluated by an untrained sensory panel (*n* = 48, ages = 18–55, males = 12, females = 36) to assess which sample (wheat‐based controls or pea‐enriched crackers) was most appreciated. The crackers containing 6% of pea flour from two different years of cultivation were presented randomly, together with a control cracker (wheat based).

The cracker samples were evaluated in terms of color, smell, taste, texture, and overall liking using a nine‐level hedonic scale, ranging from 1 to 9, as ‘very pleasant’ to ‘very unpleasant’. Purchase intention was also assessed, with nine levels ranging from ‘I would definitely buy’ to ‘I would definitely not buy’. The tests were carried out in a standardized sensory analysis room, in accordance with ISO standard EN ISO 8589.^35^

### Statistical analysis

Statistical analyses were conducted using Statistica 6.0 software (2001, StatSoft, Tulsa, OK, USA). A two‐way analysis of variance (ANOVA) and Tukey's honest significant difference test were performed to compare the growing environments with the cultivation years. Significant differences between means were determined by least significant difference values for *P* < 0.05. Pearson's correlation coefficient (r) was calculated at a significance level of *P* < 0.01.

## RESULTS

The nutritional parameters measured in this study were the phenolic compound content (polyphenols and flavonoids), antioxidant activity (DPPH and FRAP), fibers, lipids, proteins, digestible and resistant starch, carbohydrates and RFOs. Baked products (crackers) enriched with 6% organic pea flour were developed to promote the cultivation of leguminous crops, addressing the growing market demand for PBMAs. Functional (rheology and taste) and nutritional analyses were also carried out on these products, aimed at the revalorization of legume cultivation.

### Content of nutritional and bioactive compounds for location and year in pea grain

As shown in Table 2, pea grain cultivated in 2021 contained significantly higher levels of total polyphenols (TP) and total flavonoids (TF), with 1.28 mg GAE g^−1^ and 0.54 mg CE g^−1^, respectively, compared to the 2022 levels of 0.94 mg GAE g^−1^ and 0.35 mg CE g^−1^. Secondary metabolites responsible for FRAP antioxidant activity showed the same trend of TP and TF, displaying significantly higher levels in 2021 than 2022 (2.72 and 2.16 mg Fe^2+^ g^−1^, respectively); while, for DPPH, no significant differences were observed between the factors.

**Table 2:** Mean values of nutritional, anti‐nutritional (RAF and GLCT) and health‐promoting compounds of dry weight in the examined peas.

u.m.	TP	TF	FRAP	DPPH	IDF	SDF	LIP	PRO	TDS	RS	GLU	SUC	FRU	RAF	GLCT
	mg GAE g^−1^	mg CE g^−1^	mg Fe^2+^ g^−1^	μmol TE g^−1^	g kg^−1^										
Environment															
Mountainous	1.17 **a**	0.46 **a**	2.58 **a**	2.55 **a**	273.22 **a**	88.49 **a**	19.72 **a**	206.90 **b**	259.97 **a**	77.56 **a**	0.93 **b**	63.77 **a**	1.26 **a**	8.65 **a**	0.59 **a**
Hilly	1.04 **a**	0.43 **a**	2.27 **a**	3.07 **a**	289.51 **a**	90.22 **a**	22.30 **a**	239.02 **a**	233.78 **b**	67.42 **a**	1.77 **a**	47.35 **b**	2.27 **a**	11.47 **a**	0.79 **a**
Year															
2021	1.28 **a**	0.54 **a**	2.72 **a**	2.91 **a**	271.16 **a**	78.19 **b**	20.42 **a**	227.96 **a**	250.89 **a**	67.91 **a**	1.66 **a**	66.61 **a**	2.09 **a**	5.50 **b**	0.39 **b**
2022	0.94 **b**	0.35 **b**	2.16 **b**	2.70 **a**	291.57 **a**	100.51 **a**	21.59 **a**	217.97 **a**	242.86 **a**	77.08 **a**	1.03 **a**	44.51 **b**	1.45 **a**	14.62 **a**	0.99 **a**
LSD 0.05	28.51	6.70	0.15	0.99	3.79	1.35	0.34	1.21	1.57	1.47	0.07	0.85	0.11	0.52	0.023
E × Y	ns	ns	ns	ns	ns	**	ns	ns	ns	ns	ns	ns	ns	ns	ns

*Note*: Different letters within each column show significantly different values (*P* ≤ 0.05, Tukey's least significant difference test). The number of stars represent significant differences at the 0.05 (*), 0.01 (**), and 0.001 (***) probability level, respectively.

Abbreviations: CE, catechin equivalent; DPPH, 1,1‐diphenyl‐2‐picrylhydrazyl anti‐radical activity; E × Y, Environment × Year; FRAP, ferric reducing antioxidant potential; FRU, fructose content; GAE, gallic acid equivalent; GLCT, galactose content; GLU, glucose content; IDF, insoluble dietary fiber; LIP, lipids; ns, not significant; PRO, proteins; RAF, raffinose content; RS, resistant starch; SDF, soluble dietary fiber; SUC, sucrose content; TDS, total digestible starch; TE, Trolox equivalent; TF, total flavonoids; TP, total polyphenols.

In 2022 samples, SDF content resulted significantly higher (100.51 g kg^−1^) than 2021 grain (78.19 g kg^−1^), and it was observed that a correlation effect exists (*P* < 0.01) between the environmental parameters in terms of rainfalls and temperatures in the two cropping years.

As regards protein (PRO) content (Table 2), the PRO results were in line with previous literature,^36^ and the highest values of PRO were observed in hilly samples (239.02 g kg^−1^). No difference was found between the two cultivation years.

For total digestible starch (TDS) content, higher results were observed in mountainous cultivated grain (259.97 g kg^−1^), showing an inverse relationship with PRO in peas cultivated in the same environment, as demonstrated by Daba.^37^ The same trend was observed for resistant starch (RS).^38^

No significant differences in lipid (LIP) content, insoluble digestible fibers (IDF), or resistant starch (RS) were observed between the two growing environments over the 2 years of cultivation. In terms of the sucrose (SUC) content, significantly higher levels were observed in mountainous harvested grain (63.77 g kg^−1^), which is in line with the literature.^6^ However, significantly lower SUC content was evident in 2022 pea grain compared with 2021 grain (44.51 and 66.61 g kg^−1^, respectively). Finally, Table 2 shows that RFO compounds and precursors, raffinose and galactose (RAF and GLCT), were significantly higher in 2022 pea grain (5.50 and 0.39 g kg^−1^, respectively).

### Effects of weather parameters on nutritional profile of pea grain

As Table 3 shows, an inverse relationship was evident between temperature and the expression of TP, TF, and FRAP. This is in line with earlier findings.^39^ Secondary metabolites (not phenolics) responsible for DPPH antioxidant activity are involved in plant protection from abiotic stresses, and correlate significantly and positively with temperatures and negatively with rainfall during the vegetative growth of the crop.

**Table 3:** Pearson correlation between weather parameters (cumulative rainfall, mean temperature) and the nutritional profile (TP, TF, FRAP, DPPH, IDF, SDF, LIP, PRO, TDS, RS, GLU, SUC, FRU, RAFF, GLCT) in pea grain.

	Rainfall (mm)	Temperatures (°C)
TP	0.12	−0.47
TF	−0.27	−0.20
FRAP	0.11	−0.33
DPPH	−0.55*	0.52*
IDF	−0.37	0.62*
SDF	−0.09	0.44
LIP	−0.85**	0.99***
PRO	−0.98**	0.81**
TDS	0.74*	−0.79**
RS	0.83**	−0.50*
GLU	−0.69*	0.37
SUC	0.50*	−0.84**
FRU	−0.46	0.22
RAFF	−0.20	0.62*
GLCT	−0.22	0.64*

*Note*: The number of stars represent significant differences at the 0.05 (*), 0.01 (**), and 0.001 (***) probability level.

Abbreviations: TP, total polyphenols; TF, total flavonoids; FRAP, ferric reducing antioxidant potential; DPPH, 1,1 diphenyl‐2‐picrylhydrazyl anti‐radical activity; IDF, insoluble dietery fiber; SDF, soluble dietary fiber; LIP, lipids; PRO, proteins; TDS, total digestible starch; RS, resistant starch; GLU, glucose content; SUC, sucrose content; FRU, fructose content; RAF, raffinose content; GLCT, galactose content.

The LIP and PRO content showed a significant positive correlation with environment temperatures, but significant negative relationships were found with rainfall.

Conversely, a significant inverse relationship was observed between TDS, RS, and SUC content and temperatures; significant a significant positive correlation was found between these variables and rainfalls.

As regards RFOs, raffinose and galactose (RAFF and GLCT) content was analyzed and a significant and positive correlation with temperature was observed.

### Micronutrient content by location and year in pea grain

From the perspective of micronutrient composition (Table 4), peas harvested from the two growing environments (mountainous and hilly) over the two cultivation years (2021 and 2022) showed that the environment influenced micronutrient content only for K, Ca, and P concentrations. Specifically, K was the most abundant element, ranging from 9054.67 to 9395.92 mg g^−1^, whereas P ranged from 4404.87 to 5007.72 mg g^−1^. All pea samples contained higher levels of K and P than other minerals. Peas grown in the hilly (spring‐sown) environment exhibited significantly higher K, Ca, and P content (9395.92, 937.10, and 5007.72 mg g^−1^, respectively) in comparison with those from the mountainous environment. Peas from 2021 also had higher Na and Ca content (55.61 and 891.05 mg g^−1^, respectively), whereas the 2022 samples had higher K content (9337.54 mg g^−1^).

**Table 4:** Mineral composition (mg g^−1^) of dry weight in peas cultivated in the two growing environments over two cultivation years.

	Na	K	Ca	Mg	P	S	Fe	Cu	Zn
Environment									
Mountainous	54.60 **a**	9054.67 **b**	719.48 **b**	1369.43 **a**	4404.97 **b**	1865.82 **a**	60.23 **a**	12.43 **a**	47.98 **a**
Hilly	52.45 **a**	9395.92 **a**	937.10 **a**	1390.75 **a**	5007.72 **a**	1975.45 **a**	65.14 **a**	12.02 **a**	52.04 **a**
Year									
2021	55.61 **a**	9113.05 **b**	891.05 **a**	1328.59 **b**	4687.64 **a**	1913.47 **a**	66.09 **a**	11.80 **a**	50.14 **a**
2022	51.44 **b**	9337.54 **a**	765.53 **b**	1431.58 **a**	4725.05 **a**	1927.80 **a**	59.28 **a**	12.66 **a**	49.89 **a**
LSD 0.05	0.40	15.62	5.28	4.12	56.82	17.90	0.91	0.10	0.65
E × Y	ns	ns	ns	**	ns	ns	ns	*	ns

*Note*: Different letters within each column show significantly different values (*P* ≤ 0.05, Tukey's least significant difference test). The number of stars represent significant differences at the 0.05 (*), 0.01 (**), and 0.001 (***) probability levels, respectively.

Abbreviations: E × Y, environment × year; ns, not significant.

### Incorporation of organic pea in wheat‐based baked snacks (crackers)

The dough and the crackers were analyzed in terms of rheology, color, texture, nutritional composition, and microelement concentrations in both dough and final products. Sensory analysis was also performed on the final products.

### Physical characteristics of dough

**Figure 1:**
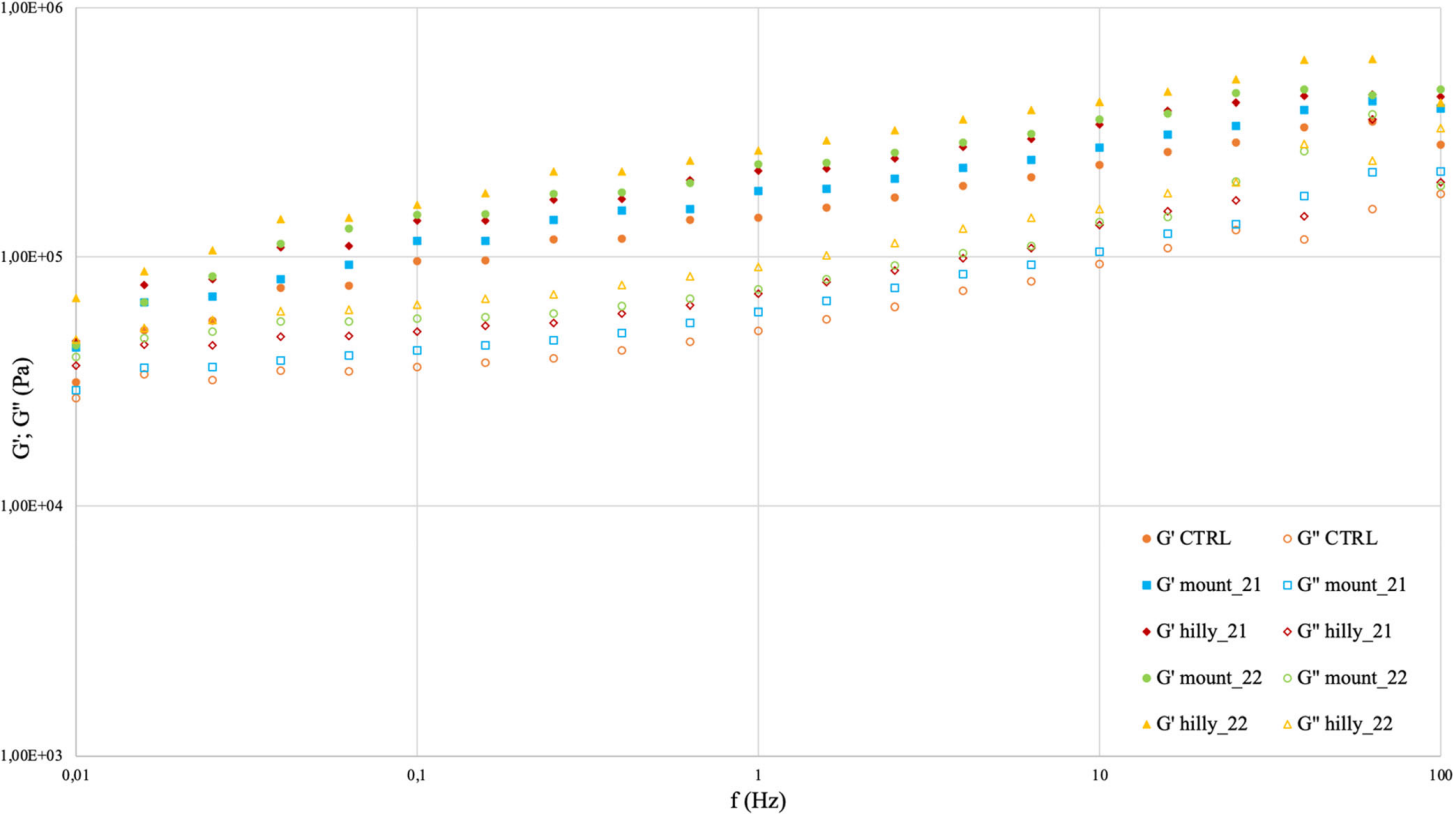
Mechanical spectra of control (CTRL) and crackers doughs prepared with 6% organic pea flour derived from two different environments (‘mountainous’ and ‘hilly’) in two cultivation years (2021 and 2022). Closed symbols represent G' (elastic modulus) and open symbols represent G" (viscous modulus).

Figure 1 presents the viscoelastic behavior of pea‐enriched cracker doughs. All doughs exhibited a mixture of elastic and viscoelastic  components, with a certain rigidity that contributed to the elastic modulus (G′), which was consistently greater than the viscous modulus (G′′) across the selected frequency range (0.1 to 100 Hz). Both G′ (storage modulus) and G′′ (loss modulus) increased with frequency for all the analysed samples. In addition, the mechanical spectra revealed that pea‐enriched samples displayed higher G′ and G′′ values compared to the wheat‐based control.

The mechanical spectra in Fig. 1 show the values for G′ and G′′ at 1 Hz (Table 5). At this frequency, both G′ and G′′ were significantly higher in pea‐enriched samples than in the control.

**Table 5:** Viscoelastic properties (G′ [Pa], G′′ [Pa] at 1 Hz) of controls and pea‐enriched doughs.

	G' 1 Hz (Pa)	G" 1 Hz (Pa)
Environment		
CTRL	1.51E+05 **b**	5.25E+04 **b**
Mountainous	2.05E+05 **a**	6.77E+04 **a**
Hilly	2.37E+05 **a**	7.84E+04 **a**
Year		
CTRL	1.51E+05 **b**	5.25E+04 **b**
2021	2.07E+05 **a**	6.86E+04 **a**
2022	1.25E+05 **a**	7.75E+04 **a**
*E × Y*	ns	ns

*Note*: Different letters within each column mean significantly different values (*P* ≤ 0.05, Tukey's least significant difference test). The number of stars represent significant differences at the 0.05 (*), 0.01 (**), and 0.001 (***) probability level, respectively.

Abbreviations: *E* × *Y*, environment × year; ns, not significant.

### Color analysis of dough

Table 6 shows the color parameters of the cracker dough. The color variations between the control and pea‐enriched doughs, expressed in *Δ*E values, were greater than 3 for all doughs, indicating that the total color difference was perceptible to the human eye. Regarding the lightness parameter (L*), increasing addition of pea flour to doughs led to a significant reduction in lightness. Regarding the color parameter a*, which measures the range between green (−60) and red,^40^ the wheat‐based control snack showed a small positive low value (0.14), while at all the pea‐enriched samples the color changed into a significantly darker green (between −3.85 and −4.80). Regarding the b* parameter, which indicates the range between blue and yellow, compared with the control, a significant (*P* < 0.05) increase was also observed with the addition of organic pea to the dough.

**Table 6:** The *Δ*E, L*, a*, b* values for control and pea‐enriched doughs.

	L*	a*	b*	*Δ*E
Environment				
CTRL	74.89 **a**	0.14 **a**	17.80 **c**	
Mountainous	66.99 **c**	−4.48 **c**	23.97 **a**	11.04
Hilly	68.38 **b**	−4.17 **b**	23.15 **b**	9.46
Year				
CTRL	74.89 **a**	0.14 **a**	17.80 **c**	
2021	68.25 **b**	−3.85 **b**	23.36 **a**	9.54
2022	67.12 **c**	−4.80 **c**	23.76 **a**	10.97
LSD 0.05	1.40	0.26	0.91	
E × Y	ns	ns	ns	

*Note*: Different letters within each column mean significantly different values (*P* ≤ 0.05, Tukey's least significant difference test). The number of stars represent significant differences at the 0.05 (*), 0.01 (**), and 0.001 (***) probability levels, respectively.

Abbreviations: E × Y, environment × year; ns, not significant.

### Physical properties of crackers

The physical properties of baked snacks frequently determine their attractiveness and desirability (or undesirability). As such, color and texture parameters (Table 7) of all crackers, with and without pea flour, were evaluated.

**Table 7:** The *Δ*E, L*, a*, b* values and texture properties for control and pea‐enriched crackers.

u.m.	Cracker color				Cracker texture	
	L*	a*	b*	*Δ*E	Hardness	Brittleness
					N	mm
Environment						
CTRL	74.14 **a**	0.96 **a**	19.17 **b**		9.13 **b**	0.68 **a**
Mountainous	72.76 **a**	−2.71 **b**	21.83 **a**	4.74	11.63 **a**	0.70 **a**
Hilly	73.25 **a**	−2.56 **b**	21.37 **a**	4.25	10.74 **ab**	0.92 **a**
Year						
CTRL	74.14 **a**	0.96 **a**	19.17 **b**		9.13 **b**	0.68 **a**
2021	72.45 **a**	−2.17 **b**	21.92 **a**	4.50	11.78 **a**	0.83 **a**
2022	73.55 **a**	−3.10 **c**	21.27 **a**	4.62	10.51 **a**	0.81 **a**
LSD 0.05	2.13	0.60	1.33		2.35	0.35
E × Y	ns	***	***		ns	*

*Note*: Different letters within each column mean significantly different values (*P* ≤ 0.05, Tukey's least significant difference test). The number of stars represent significant differences at the 0.05 (*), 0.01 (**), and 0.001 (***) probability level, respectively.

Abbreviations: E × Y, environment × year; ns, not significant.

**Figure 2:**
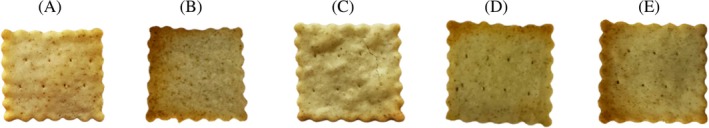
Representative images of controls and crackers prepared with 6% of organic pea flour derived from two different environments over two cropping years (2021 and 2022). A = wheat‐based control, B = mountainous 2021, C = hilly 2021, D = mountainous 2022, E = hilly 2022.

Regarding the color parameters, pea‐enriched crackers showed significantly lower a* levels (ranging between −2.17 to −3.10) in comparison with the control (0.96) and the color intensity resulted in a slightly green appearance compared with the previously discussed doughs. As with doughs, increasing addition of pea flour to crackers resulted in visually different crackers (*Δ*E > 3), as can be seen in Fig. 2.

The texture results (Table 7) indicate that variations observed in the dough are reflected in the final products. Crackers made with pea flour exhibited higher hardness (ranging from 10.51 to 11.78 N) compared to the wheat‐based control (9.13 N). This trend was also observed in the brittleness parameter, where pea‐enriched crackers required a greater distance to reach the maximum peak, although no significant differences were found.

### Nutritional properties of crackers

The proximate analysis of foods involves the determination of the principal components, such as moisture, ash (total minerals), fats and proteins. Water activity (a_w_) and bioactive compounds (phenolics) and relative antioxidant activity were also evaluated. Table 8 presents the proximate composition of the crackers prepared with pea flour compared with the wheat‐based control.

**Table 8:** Functional and nutritional composition of control and pea‐enriched crackers.

	a_w_	Moisture	Ash	Fats	PRO	TP	TF	FRAP	DPPH
	u.m.	%			g kg^−1^	mg GAE g^−1^	mg CE g^−1^	mg Fe^2+^ g^−1^	μmol TE g^−1^
Environment									
CTRL	0.12 **c**	0.72 **c**	1.79 **b**	10.5 **ab**	96.59 **b**	609.31 **b**	2330.43 **b**	1.22 **b**	0.83 **b**
Mountainous	0.25 **b**	3.21 **b**	2.39 **a**	12.47 **a**	103.33 **a**	801.37 **a**	2913.12 **a**	1.41 **a**	1.03 **a**
Hilly	0.28 **a**	4.24 **a**	2.02 **b**	9.85 **b**	103.82 **a**	642.12 **b**	2886.35 **a**	1.19 **b**	0.68 **b**
Year									
CTRL	0.12 **c**	0.72 **b**	1.79 **b**	10.5 **b**	96.59 **b**	609.31 **b**	2330.43 **b**	1.22 **b**	0.83 **ab**
2021	0.23 **b**	3.40 **a**	2.30 **a**	13.85 **a**	104.14 **a**	693.31 **a**	2933.96 **a**	1.33 **a**	1.01 **a**
2022	0.29 **a**	4.04 **a**	2.10 **ab**	8.47 **b**	103.00 **a**	75.018 **a**	2865.51 **a**	1.27 **ab**	0.70 **b**
LSD 0.05	1.04	0.48	0.011	2.36	0.19	8.21	17.97	0.04	0.21
E × Y	***	ns	***	ns	ns	*	***	ns	**

*Note*: Different letters within each column mean significantly different values (*P* ≤ 0.05, Tukey's least significant difference test). The number of stars represent significant differences at the 0.05 (*), 0.01 (**), and 0.001 (***) probability levels, respectively.

Abbreviations: CE, catechin equivalent; DPPH, 1,1‐diphenyl‐2‐picrylhydrazyl anti‐radical activity; E × Y, environment × year; FRAP, ferric reducing antioxidant potential; GAE, gallic acid equivalent; ns, not significant; PRO = proteins; TE, Trolox equivalent; TF, total flavonoids; TP, total polyphenols.

Pea‐enriched crackers exhibited significantly higher PRO content than the control, as expected, showing PRO values ranging between 103.00 to 104.14 g kg^−1^ in comparison with wheat‐based products (96.59 g kg^−1^). However, no significant differences between the environments and the cultivation years were observed.

Mountainous and 2021 samples displayed significantly higher ash content (2.39 and 2.30%, respectively). On the other hand, the highest a_w_ levels were observed in hilly and 2022 samples (0.28 and 0.29, respectively), with the lowest result for controls (0.12). The same trend was observed for moisture content.

The total flavonoids (TF) showed overall higher levels in pea‐enriched samples compared to the controls, both from environment and cultivation year perspectives. In particular, TF concentration in pea‐enriched samples ranged between 2865.51 and 2913.12 mg g^−1^. For the other nutritional components (TP, FRAP, and DPPH assay), this trend was not observed. Crackers from the hilly environment were found to be similar to the wheat‐based products. The pea‐based crackers exhibited TP values ranging from 609.31 to 642.12 mg g^−1^, FRAP values between 1.19 and 1.33 mg Fe^2+^ g^−1^, and DPPH values between 0.68 and 1.03 μmol TE g^−1^. However, for these compounds the concentration of the pea‐enriched crackers in the two cultivation years (2021 and 2022) was significantly higher than the wheat‐based control.

The mineral profile results are presented in Table 9. According to the literature, pure pea flour is a source of K, P, Mg, and Cu,^41^, ^42^ producing similar properties in crackers containing it. However, in terms of K, P, Mg, and Zn significant higher content in pea‐enriched crackers was observed compared to wheat‐based controls.

**Table 9:** Mineral composition (mg g^−1^) of control and pea‐enriched crackers.

	Na	K	Ca	Mg	P	S	Fe	Cu	Zn
Environment									
CTRL	9498.57 **a**	1744.00 **b**	192.25 **a**	228.77 **b**	2991.04 **c**	32.17 **a**	995.77 **a**	3.28 **a**	6.75 **b**
Mountainous	9297.39 **a**	2486.06 **a**	234.26 **a**	328.27 **a**	3183.02 **b**	34.77 **a**	1024.41 **a**	3.53 **a**	10.49 **a**
Hilly	9605.02 **a**	2436.73 **a**	221.70 **a**	324.98 **a**	3274.71 **a**	32.15 **a**	1023.13 **a**	3.42 **a**	10.20 **a**
Year									
CTRL	9498.57 **ab**	1744.00 **b**	192.25 **a**	228.77 **b**	2991.04 **b**	32.17 **a**	995.77 **a**	3.28 **a**	6.75 **b**
2021	9207.26 **b**	2426.70 **a**	227.92 **a**	323.37 **a**	3229.65 **a**	32.17 **a**	1021.97 **a**	3.34 **a**	10.47 **a**
2022	9695.15 **a**	2496.10 **a**	228.04 **a**	329.89 **a**	3228.07 **a**	34.76 **a**	1025.57 **a**	3.60 **a**	10.22 **a**
LSD 0.05	45.44	13.28	4.90	1.73	8.96	0.63	5.61	0.046	0.064
E × Y	*	ns	ns	ns	ns	ns	ns	ns	ns

*Note*: Different letters within each column indicate significantly different values (*P* ≤ 0.05, Tukey's least significant difference test). The number of stars represent significant differences at the 0.05 (*), 0.01 (**), and 0.001 (***) probability levels, respectively.

Abbreviations: E × Y, environment × year; ns, not significant.

### Sensory analysis of crackers

**Figure 3:**
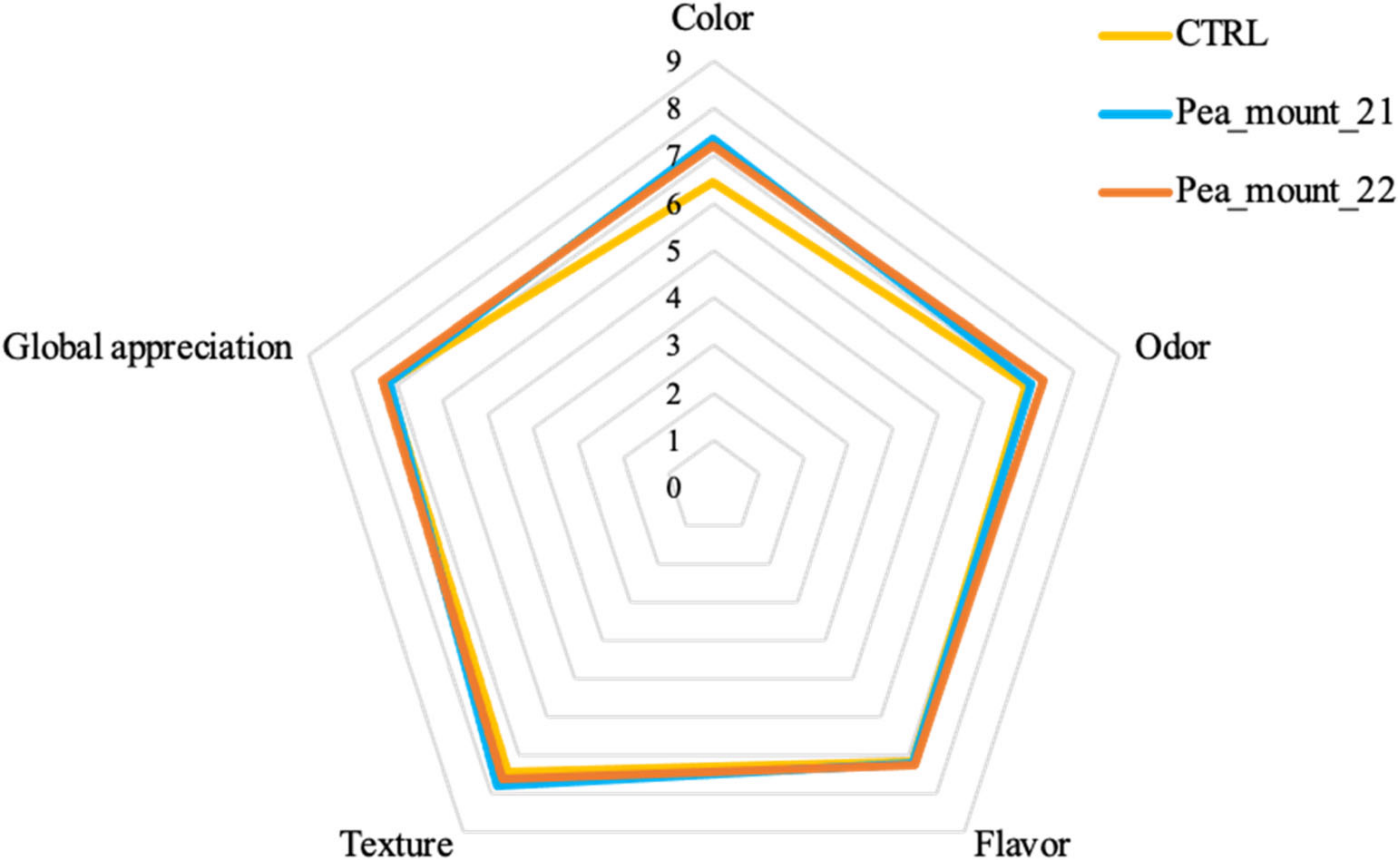
Sensory profile of baked snacks: evaluation of sensory attributes (color, appearance, aroma, texture, and flavor) and global appreciation. Values are presented as means.

Sensory analysis was carried out on one wheat‐based control and two pea‐enriched crackers, both from mountainous environments but in two different cultivation years (2021 and 2022) for a total of three samples. Evaluation of the sensory attributes of snacks (Fig. 3) revealed that color parameter was significantly (*P* < 0.05) differently perceived by the sensory panel, between the pea‐enriched crackers and the control. In general, both pea‐enriched and wheat‐based (control) snacks presented the most desirable sensory attributes (color, odor, flavor, texture, and global appreciation) with no significant differences.

Regarding purchase intentions (data not shown), no significant differences were observed between all the three samples (control and pea flour from mountainous environment during 2021 and 2022).

## DISCUSSION

Peas are a rich source of various bioactive compounds. Previous studies, although scarce, have shown that environment conditions may affect the nutritional composition of legumes, in particular peas.^43^ More recent studies have also showed variations in health‐promoting compounds based on location.^38^, ^44^, ^45^

Therefore, a multidisciplinary approach was used to study how different crop‐growing locations in Emilia Romagna (Italy), as well as different agronomic practices, such as sowing time (autumn and spring), may affect the nutritional composition of the harvested grain over two consecutive cultivation seasons (2021 and 2022).

### Nutritional and bioactive compound content by location and year in pea grain

Leguminous plants play a major role in human nutrition and are a good source of saccharides, proteins, micronutrients and bioactive compounds.^46^ Phenolic acids are the principal polyphenols found in grains and pulses; they primarily exist as bound derivatives, in particular as conjugates with polysaccharides and proteins.^47^ Previous studies have shown that the content of some phenolics may increase when certain stress conditions are applied such as UV radiation, infection by pathogens and parasites, wounding, air pollution, and exposure to extreme temperatures.^48^, ^49^ Hence, location and year of cultivation (weather and environment conditions) may influence these bioactive compounds' concentration and relative antioxidant activity in the plant fruit.

In this study, the higher levels of TP, TF, and FRAP antioxidant activity of 2021 peas among the two growing environments (mountainous and hilly) may reflect plant response to specific abiotic stresses on both growing environments during 2021;^39^ in particular the average annual rainfall and temperature conditions displayed lower levels in 2021 (299.8 mm and 14.56 °C, respectively) than 2022 (344.7 mm and 16.91 °C, respectively), showing that drought and cold conditions evidently stimulated the biosynthesis of phenolic compounds and relative antioxidant activity in the resulting plants and/or grains.

The significant interaction (E × Y) for SDF content suggests that the year of cultivation was strongly influenced by the growing environment. This should be further investigated in future studies.

As regards pea protein (PRO), a previous study^43^ based on 19 pea cultivars cultivated in four different locations over 3 years found a significant correlation between protein content and location, emphasizing the importance of environment and growing conditions and the cultivation year for grain nutritional composition. More recent studies have also confirmed the importance of environment for the protein content in peas.^45^, ^50^, ^51^, ^52^

In this study, the highest PRO results were found in hilly grown peas, suggesting that spring environment conditions support the accumulation of proteins during the vegetative growth of the plant. Moreover, a study by Tao^38^ found that extreme weather conditions (in terms of rainfall and temperature) during sowing and the vegetative growth of the plant had a strong impact on pea protein content, thus supposing better overall plant health conditions in hilly environments.

In contrast with PRO, starch (TDS) content showed to be higher in mountainous grain. Indeed, a study conducted by Mohammed^6^ stated that drier and warmer environmental conditions (65.6 and 48.7 mm of cumulative rainfalls for hilly and mountainous environment, respectively) during the crop's reproductive phase until seed development stimulate the accumulation of starch in pea grain, due to rapid conversion of sugars into starch within the grain. The same trend was observed for RS,^38^ and this gained attention recently.^53^, ^54^ It is a component of starch that, during the process of digestion, is fermented in the colon^54^ and may help in controlling diabetes and energy balance, and the short‐chain fatty acids produced by fermenting colonic bacteria provide direct health benefits to the colon.^55^

As regards sugars, an inverse relationship can be noticed between the SUC and RFO content in the examined samples. Raffinose family oligosaccharides (RFOs) are considered as anti‐nutritional compounds because they are believed to be responsible for causing flatulence in humans, which is the most important factor in deterring people from including more legumes in their diet. It was found that RFO biosynthesis occurs in the latter stages of seed development and is strictly dependent on SUC content.^56^ It was shown that sucrose plays an active role in the first steps of raffinose synthesis^57^ and it is also a source of UDP‐glucose, which is involved in the galactose (RFO precursor) biosynthetic pathway.^58^ It can therefore be assumed that elevated concentrations of SUC can stimulate the RFOs pathway by increasing the effectiveness of metabolic activity of specific enzymes.

### Effects of weather parameters on the nutritional profile of pea grain

To further investigate the impact that growing environment and cultivation year exert on the nutritional profile of legumes, more in particular in pea grain, the relationship between nutritional compounds and weather conditions was correlated (Table 3).

The LIP and PRO content correlated positively with temperature and negatively with rainfall. Sattari Vayghan^59^ found that lipid content was highly dependent on seasonal temperatures, which can induce structural modifications at high temperatures. However, in both growing environments, peas were harvested before reaching this thermal threshold, confirming the positive correlation with temperature.

Regarding PRO, earlier studies by Karjalainen and Kortet^60^ and more recent work by Tao^38^ demonstrated that excessive moisture or drought during seed development can limit protein accumulation in peas, supporting the inverse relationship between protein content and rainfall observed in this study.

For starch and sugars, Mohammed^6^ and Tao^38^ investigated the behavior of TDS, RS, and SUC, showing their strong dependence on temperature during the plant's vegetative and reproductive stages, as well as seed development. Similarly, RFOs (RAFF and GLCT) exhibited a positive correlation with temperature, likely due to their inverse relationship with SUC, which plays an active role in the biosynthesis of these anti‐nutritional compounds.^56^, ^57^

Overall, in terms of the nutritional profile of organic peas cultivated in different locations of the Emilia‐Romagna region, all the nutritional and bioactive compounds are interconnected and, interestingly, the weather parameters exhibited a strong impact on them. Hence, it can be confirmed that growing environment conditions may impact the nutritional composition and thus improve the quality of the grain, to encourage global consumer interest through high‐quality food products in a more sustainable food system.

### Micronutrient content by location and year in pea grain

Among the micronutrients listed in Table 4, environmental conditions showed no significant impact on most components, except for K, Ca, and P, which were higher in samples grown in hilly locations. This suggests that spring weather conditions during vegetative growth of the crop may have positively influenced plant health and its grain development.

In a study by Hacisalihoglu^61^ was found a positive correlation between Ca and Mg was found, and similarly with Cu, P, K and S, suggesting that qualitative content in peas could be improved by targeting either Ca or Mg. However, several studies have reported that environmental factors displayed a strong impact on mineral content in peas,^40^, ^62^, ^63^ and, in particular, Wang^40^ found that Cu and Zn showed significant differences based on the environmental conditions of the vegetative growth of the plant, and the opposite behavior was observed for Mn or P content; nevertheless, on the whole, results suggest that genetics are the driving factor for the accumulation of minerals in grain legumes.^61^

### Incorporation of organic pea in wheat‐based crackers

To encourage legume production for a more sustainable agronomic system and to respond to the growing consumer demand for more plant‐based protein alternatives to meat, the preparation of savory baked products (crackers) enriched with organic pea flour was set up. To assess the impact of pea incorporation on the technological properties of the dough and the final product, a wheat‐based control was prepared. In parallel, to investigate the impact of different locations and year of cultivation on the preparation of savory baked snacks, four pea‐enriched crackers were prepared: two different growing environments (mountainous and hilly) over two cultivation years (2021 and 2022) and compared to the wheat‐based cracker control.

### Physical characteristics of dough

Figure 1 illustrates the predominance of elastic behavior in the doughs, with greater elasticity indicating increased mechanical strength and shape retention ability.^64^ The increasing G′ and G″ values suggest a weak gel‐like rheological behavior, characteristic of cracker doughs, aligning with findings by Mota^32^ and Fradinho.^65^

The higher G′ and G″ levels in pea‐enriched samples in comparison with the wheat‐based control suggest that pea flour strengthens the dough structure by forming a stronger network between pea proteins, polysaccharides, and wheat flour compounds, as previously reported by Mohammed.^66^ Studies have shown that incorporating legumes into wheat dough increases protein water absorption, reducing water availability for gluten network development.^67^ Zucco^68^ highlighted that legume flours contain more hydrophilic sites due to their higher protein content, competing for the limited free water in dough. Sugars and fibers may also influence the formation of a structured dough matrix.^69^, ^70^

At the selected frequency (1 Hz) shown in Table 5, G′ and G″ values for pea‐enriched samples were higher than the control, indicating increased dough elasticity. This trend supports the role of pea proteins – and legume proteins in general – in dough formation and stabilization through interactions with polysaccharides and free sugars in wheat flour, as well as their higher water absorption capacity.

Regarding color parameters, the color differences between the pea‐enriched crackers and the control were distinguishable by the human eye.^71^ The high levels of plant pigment (chlorophyll) in pea‐enriched samples led to the formation of green‐colored doughs. This resulted in a significant reduction in the lightness parameter (L*) in all pea‐enriched products, promoting darkening effects.^66^, ^70^, ^71^

### Physical properties of crackers

In Table 7, pea‐enriched crackers acquired a slightly green color compared to the doughs. This is possibly due to Maillard reactions between reducing sugars and amino acids but is also possibly a result of starch dextrination and sugar caramelization.^68^, ^72^

As regards texture analysis on the snacks, pea‐enriched crackers showed higher levels of hardness than the control. The impact of the incorporation of pea in the products is related to the macromolecular structure within the pea flour, due to the absence of gluten. Indeed, Dhinda^73^ indicated that pea flour promoted a structural rearrangement leading to various interactions amongst starch and proteins (protein‐starch complex) and altered by the heat treatment that occurs during the cooking process. Moreover, as mentioned above, the water content was kept the same when replacing wheat flour (in the control) by pea flour. Increased protein‐starch interactions promote a structuring effect, enhanced by the ability of pea flour to absorb water. The same trend was also observed regarding brittleness, indicating a higher degree of crispness compared to the control snacks. Brittleness is the distance (in mm) to reach the maximum breaking peak and is considered to be an indicator of crispness.

From these findings, it can be concluded that crackers incorporating pea flour may appear more attractive to consumers.

### Nutritional properties of crackers

Several studies observed increases in a_w_ values with the addition of apple fibers to cookies^74^ and with the addition of microalgae with high protein content.^75^ Similar behavior was also found for pea‐enriched crackers. It can thus be assumed that the increases in protein content led to higher water‐holding capacity values compared to the control. Due to the higher water absorption capacity of pea proteins, pea‐enriched crackers exhibited higher moisture and water activity (a_w_) levels compared to the control samples. However, pea incorporation did not affect the preservation characteristics of the final products.

The incorporation of organic pea improved the nutritional quality and bioactive compound profile of the final product, including proteins (PRO), total polyphenols (TP), total flavonoids (TF), and their associated antioxidant activity. These findings suggest that legume‐based crackers represent a sustainable and health‐conscious alternative to wheat‐based snacks, potentially increasing consumer appeal.

As regards the mineral profile of the snacks, although cooking heating treatment reduced levels of minerals,^76^ higher levels of K, P, Mg, and Zn were observed in pea‐enriched crackers than in the controls. It can be concluded that incorporation of pea flour in wheat‐based baked snacks may lead to food products with higher nutritional quality.

### Sensory analysis of crackers

Sensory analysis of selected pea‐enriched samples and the wheat‐based control (Fig. 3) indicated that color was the primary factor influencing product appreciation and quality, as supported by previous studies.^66^, ^68^, ^70^, ^77^ No significant differences were observed for key sensory attributes (color, odor, flavor, texture, and overall appreciation) or purchase intent, suggesting that pea incorporation does not compromise the product's presentation or appeal. However, higher pea flour levels (up to 20%) led to a significant reduction in taste, odor, and overall acceptance due to the intensified leguminous flavor and aroma.

Overall, the increases in the amounts of protein and in several physicochemical parameters corroborate that the addition of pea flour can increase the quality of the savory snacks produced. The use of pea in the final food product may therefore be advantageous, due to its high quantities of digestible plant protein, starch, and dietary fiber, and its particular low amounts of anti‐nutritional compounds.

## CONCLUSION

Leguminous plants represent a sustainable, nutritious, and convenient source of protein and are a promising alternative to meat. Their nutritional value, low environmental impact, and human health benefits make them a key choice in promoting sustainable diets and mitigating the negative impacts of the meat industry. In the current market, legume‐based products are becoming increasingly popular as functional foods with the use of alternative flours, providing both added nutritional value and bioactive healthy compounds.

The present study aimed to investigate the effects of agronomical and environmental conditions on the nutritional and bioactive composition of peas cultivated in different Italian environments over different cultivation years. This was an important consideration when prioritizing this crop for future research, development, and innovation. An efficient delivery method for incorporating high nutritional quality organic pea flour into baking products was presented. Nutritionally, the environment exerts a strong impact on the nutritional quality of the grain, mostly due to weather conditions during the vegetative and reproductive stage of the crop and seed development. Technologically, it was found that the incorporation of pea into wheat‐based crackers had an impact on the dough and final product in terms of functional and nutritional quality parameters. making them more visually appealing to consumers and serving as an effective vehicle for delivering a plant‐based protein source as an alternative to meat.

## CONFLICT OF INTEREST

The authors declare that they have no known competing financial interests or personal relationships that could appear to influence the work reported in this paper.

## Supporting information


**Data S1.** Supplementary Information.

## Data Availability

The data that support the findings of this study are available from the corresponding author upon reasonable request.
