# Supplementary material for: Nutritional features of organic peas (Pisum sativum L.) cultivated in different Italian environments and rheological profile of pea‐enriched crackers
Source: J Sci Food Agric. 2025 Feb 5;105(7):3606–19. doi: 10.1002/jsfa.14156 (PMC11990044; doi:10.1002/jsfa.14156)
Supplement: Supplementary file 1 — Data S1. Supplementary Information. [file JSFA-105-3606-s001.docx]

**Supplementary Material**

**Table 1.** Moisture % and Dry matter of the examined peas (*Pisum sativum* L.). Different letters withing each column show significant different values (p ≤ 0.05, Tukey’s least significant difference test). The number of stars represent significant differences at the 0.05 (*), 0.01 (**), and 0.001 (***) probability level, respectively. ns = not significant.

|  | | Moisture % | Dry matter |
| --- | --- | --- | --- |
| Environment | mountainous | 7.77 **a** | 92.53 **a** |
|  | hilly | 7.37 **a** | 92.31 **a** |
|  |  |  |  |
| Year | 2021 | 7.46 **a** | 92.23 **a** |
|  | 2022 | 7.68 **a** | 92.62 **a** |
|  |  |  |  |
|  | *LSD 0.05* | *1.58* | *0.46* |
|  | *E x Y* | *ns* | *** |

|  |  | TP | TF | FRAP | DPPH | IDF | SDF | LIP | PRO | TDS | RS | GLU | SUC | FRU | RAF | GLCT |
| --- | --- | --- | --- | --- | --- | --- | --- | --- | --- | --- | --- | --- | --- | --- | --- | --- |
|  | u.m. | mg GAE g^-1^ | mg CE g^-1^ | mg Fe^2+^ g^-1^ | μmol TE g^-1^ | g kg^-1^ | | | | | | | | | | |
| **Mountainous** | 2021 | 1.43 **a** | 0.52 **a** | 3.20 **a** | 3.27 **a** | 256.19 **a** | 89.47 **a** | 19.35 **a** | 217.21 **a** | 270.16 **a** | 83.59 **a** | 0.97 **a** | 71.17 **a** | 0.89 **a** | 5.37 **b** | 0.40 **b** |
|  | 2022 | 0.91 **b** | 0.34 **b** | 1.99 **b** | 1.82 **b** | 290.25 **a** | 87.50 **a** | 20.09 **a** | 196.60 **b** | 249.78 **a** | 71.54 **a** | 0.88 **a** | 56.37 **b** | 1.62 **a** | 11.93 **a** | 0.78 **a** |
|  | **Average** | **1.17** | **0.43** | **2.59** | **2.55** | **273.22** | **88.48** | **19.72** | **206.905** | **259.97** | **77.56** | **0.92** | **63.77** | **1.25** | **8.65** | **0.59** |
|  |  |  |  |  |  |  |  |  |  |  |  |  |  |  |  |  |
| **Hilly** | 2021 | 1.13 **a** | 0.56 **a** | 2.25 **a** | 2.55 **a** | 286.13 **a** | 66.91 **b** | 21.50 **a** | 238.70 **a** | 231.62 **a** | 64.27 **a** | 2.35 **a** | 62.04 **a** | 3.28 **a** | 5.63 **a** | 0.37 **b** |
|  | 2022 | 0.96 **a** | 0.36 **b** | 2.34 **a** | 3.58 **a** | 292.90 **a** | 113.52 **a** | 23.09 **a** | 239.34 **a** | 235.94 **a** | 70.57 **a** | 1.18 **a** | 32.65 **b** | 1.27 **a** | 17.3 **a** | 1.2 **a** |
|  | **Average** | **1.04** | **0.46** | **2.30** | **3.07** | **289.51** | **90.21** | **22.29** | **239.02** | **233.78** | **67.42** | **1.76** | **47.34** | **2.27** | **11.46** | **0.78** |
|  |  |  |  |  |  |  |  |  |  |  |  |  |  |  |  |  |
|  | **AVR 2021** | 1.28 **a** | 0.54 **a** | 2.27 **a** | 2.91 **a** | 271.16 **a** | 78.19 **b** | 20.42 **a** | 227.95 **a** | 250.89 **a** | 67.91 **a** | 1.66 **a** | 66.60 **a** | 2.08 **a** | 5.50 **b** | 0.38 **b** |
|  | **AVR 2022** | 0.94 **b** | 0.35 **b** | 2.17 **b** | 2.70 **a** | 291.57 **a** | 100.51 **a** | 21.59 **a** | 217.97 **a** | 242.86 **a** | 77.08 **a** | 1.03 **a** | 44.51 **b** | 1.44 **a** | 14.61 **a** | 0.99 **a** |

**Table 2.** Mean values of nutritional, anti-nutritional (RAF & GLCT) and health-promoting compounds of dry weight in examined peas (*Pisum sativum* L.). Different letters withing each column show significant different values (p ≤ 0.05, Tukey’s least significant difference test). The number of stars represent significant differences at the 0.05 (*), 0.01 (**), and 0.001 (***) probability level, respectively. ns = not significant, TP = total polyphenols, TF = total flavonoids, FRAP = ferric reducing antioxidant potential, DPPH = 1,1-diphenyl-2-picrylhydrazyl anti-radical activity, IDF = insoluble dietary fiber, SDF = soluble dietary fiber, LIP = lipids, PRO = proteins, TDS = total digestible starch, RS = resistant starch, GLU = glucose content, SUC = sucrose content, FRU = fructose content, RAF = raffinose content, GLCT = galactose content, GAE = gallic acid equivalent, CE = catechin equivalent, TE = Trolox equivalent and E x Y = Environment x Year.
